# Epidemiological analysis of 67 local COVID-19 clusters in Sichuan Province, China

**DOI:** 10.1186/s12889-020-09606-4

**Published:** 2020-10-08

**Authors:** Suling Mao, Ting Huang, Heng Yuan, Min Li, Xiaomei Huang, Changxiao Yang, Xingyu Zhou, Xiuwei Cheng, Qian Su, Xianping Wu

**Affiliations:** 1 Sichuan Center for Disease Control and Prevention, No. 6 Zhongxue Road, Wuhou District, Chengdu, 610,041 Sichuan China; 2 Jianyang Center for Disease Control and Prevention, Chengdu, Sichuan China; 3 Panzhihua Center for Disease Control and Prevention, Panzhihua, Sichuan China

**Keywords:** Cluster, COVID-19, Epidemiology

## Abstract

**Background:**

This study was intended to investigate the epidemiological characteristics of COVID-19 clusters and the severity distribution of clinical symptoms of involved cases in Sichuan Province, so as to provide information support for the development and adjustment of strategies for the prevention and control of local clusters.

**Methods:**

The epidemiological characteristics of 67 local clusters of COVID-19 cases in Sichuan Province reported as of March 17, 2020 were described and analyzed. Information about all COVID-19 clusters and involved cases was acquired from the China Information System for Disease Control and Prevention and analyzed with the epidemiological investigation results taken into account.

**Results:**

The clusters were temporally and regionally concentrated. Clusters caused by imported cases from other provinces accounted for 73.13%; familial clusters accounted for 68.66%; the average attack rate was 8.54%, and the average secondary attack rate was 6.11%; the median incubation period was 8.5 d; a total of 28 cases met the criteria for incubation period determination, and in the 28 cases, the incubation period was > 14 d in 21.43% (6/28). a total of 226 confirmed cases were reported in the 67 clusters. Ten cases were exposed before the confirmed cases they contacted with developed clinical symptoms, and the possibility of exposure to other infection sources was ruled out; two clusters were caused by asymptomatic carriers; confirmed cases mainly presented with fever, respiratory and systemic symptoms; a gradual decline in the severity of clinical symptoms was noted with the increase of the case generation.

**Conclusions:**

Population movement and gathering restrictions and strict close contact management measures will significantly contribute to the identification and control of cases. Transmission during the incubation period and asymptomatic infections have been noted. Studies on the pathogenicity and transmissibility in these populations and on COVID-19 antibody levels and protective effects in healthy people and cases are required.

## Background

In later December 2019, a number of pneumonia cases of unknown etiology emerged in Wuhan, China. Around the Chinese New Year in 2020, the number of cases rose sharply, involving multiple provinces, municipalities and autonomous regions in China; at the same time, outbreaks were reported in many other countries including Japan, Korea, Italy and the United States. On March 11, 2020, the WHO declared COVID-19 a pandemic [1]. On January 16, 2020, the first COVID-19 cluster was reported in Sichuan Province, China; as of March 17, 2020, a total of 67 local clusters were reported, involving 226 confirmed COVID-19 cases. Sichuan Province is a populous province in the southwest of mainland China, so absence of stringent and effective prevention and control could have resulted in serious social consequences. We analyzed the epidemiological characteristics of COVID-19 clusters in Sichuan, intended to provide information support for the development and adjustment of local prevention and control strategies.

## Methods

### Study design

The epidemiological characteristics of 67 local COVID-19 clusters in Sichuan reported as of March 17, 2020 and confirmed cases involved in these clusters were described and analyzed using a cross-sectional study design.

### Data source

In accordance with requirements in the *Guidance for Corona Virus Disease 2019: Prevention, Control, Diagnosis and Management* [2], information about all COVID-19 clusters and cases should be reported to the China Information System for Disease Control and Prevention. In this study, information about all local clusters was exported from sub-module “Emergency Public Reporting System” and data about related confirmed cases and asymptomatic carriers from sub-module “Infectious Disease Management Information System”. Based on epidemiological investigation results, transmission chains were constructed and case generations were determined, followed by a comprehensive analysis. This study has covered all local clusters and all confirmed cases and asymptomatic carriers in the clusters, so sampling was not involved. Confirmed cases with missing clinical information were not included into the analysis.

### Definitions

Confirmed cases, asymptomatic carriers and clusters were identified according to the definitions in the *Guidance for Corona Virus Disease 2019: Prevention, Control, Diagnosis and Management* [2]. A cluster outbreak indicated that more than two confirmed cases or asymptomatic carriers were found within 14 days in a small area (such as a family, a building site, a work unit), and there was a possibility of human-to-human transmission caused by close contact or by exposure to infectious source together. All confirmed cases were divided into mild cases (the clinical symptoms are mild and no pneumonia manifestation can be found in imaging); ordinary cases (with symptoms like fever and respiratory tract symptoms, and pneumonia manifestation can be seen in imaging); and severe cases (respiratory distress, RR ≥ 30 breaths/min; pulse oxygen saturation (SpO2) ≤ 93% on room air at rest state; arterial partial pressure of oxygen (PaO2)/oxygen concentration (FiO2) ≤ 300 mmHg. The date of onset for a confirmed case was defined as the date of first appearance of clinical symptoms self-reported in field epidemiological investigation. The date of onset for an asymptomatic carrier was defined as the date on which a positive COVID-19 pathological test was obtained with respiratory tract or other feasible samples.

The attack rate was defined as the number of cases divided by the number of exposed persons, where the number of cases was the total number of confirmed cases involved in a transmission chain. As both second-generation (G2) and third-generation (G3) cases were identified from close contacts of cases of the previous generations, if any G2 case developed in a cluster, the number of exposed persons was the number of close contacts of first-generation (G1) cases plus the number of G1 cases; if any G3 case developed in a cluster, the number of exposed persons was the number of close contacts of G1 cases plus the number of close contacts of G2 cases resulting in the G3 cases plus the number of G1 cases; and so forth. The secondary attack rate was defined as the number of second- and later-generation cases divided by the number of exposed persons, where the number of exposed persons was calculated using the aforementioned algorithm except that the number of G1 cases was not included. The incubation period was determined based on confirmed cases that had been exposed for a single time in transmission chains, whose exposure and onset time were clearly known and in whom other factors potentially responsible for their infection were ruled out. Cases for the determination of the attack rate, the secondary attack rate and the incubation period included confirmed cases and asymptomatic carriers. A family dinner was defined as a dinner attended by two or more families.

### Statistical analyses

Epidemiological characteristics of clusters and demographic and clinical characteristics of confirmed cases were descriptively analyzed. Attack rates, secondary attack rates and clinical types of cases and constituent ratios of symptoms were compared using *χ*2 test for constituent differences. The regional distribution map in Fig. 2 was created by geocoding all COVID-19 clusters and matching them to the city-level layers of polygon and point by administrative codes with the use of ArcGIS software.

## Results

### Distribution of clusters

**Fig. 1:**
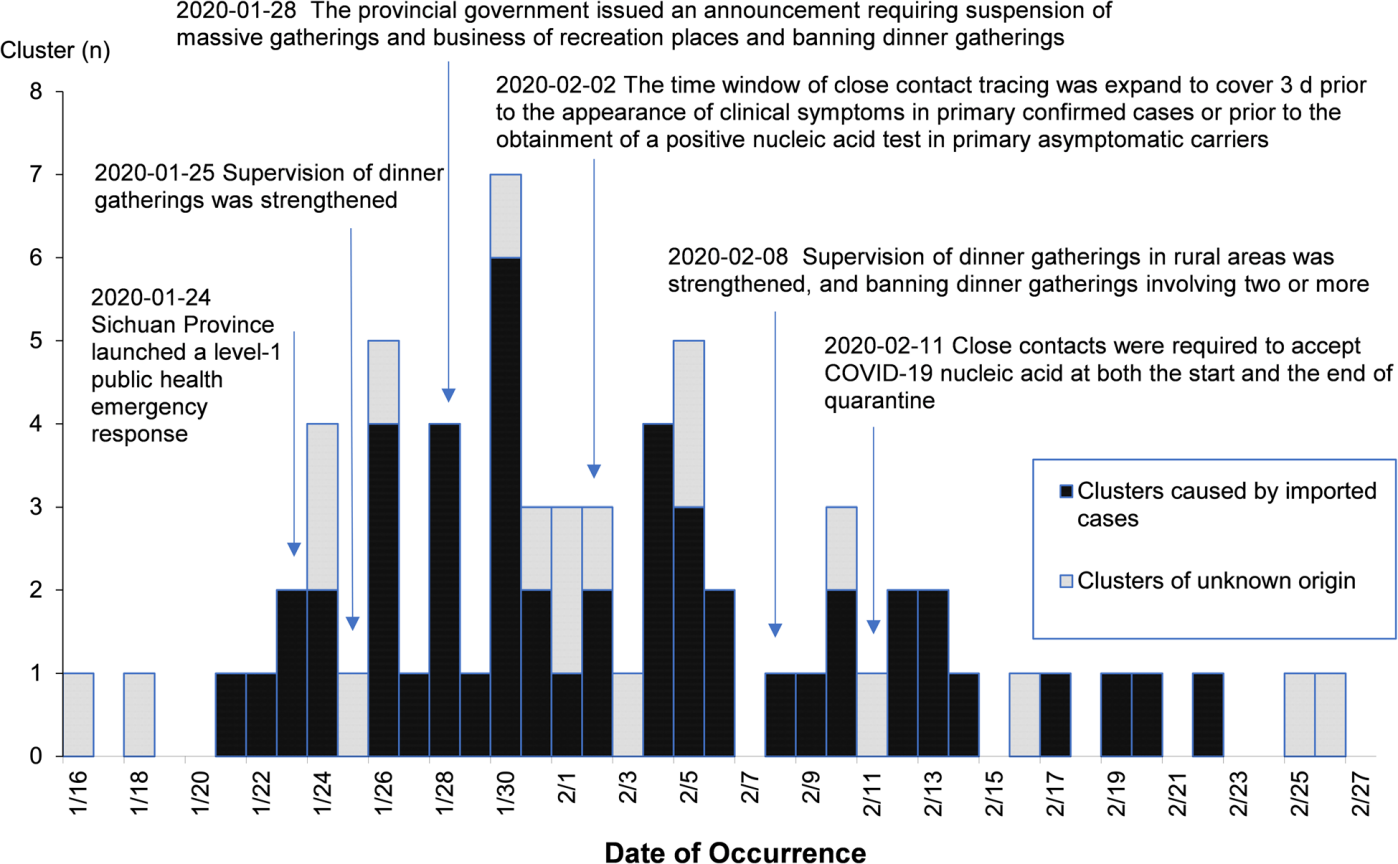
Temporal distribution of cluster reports (*n* = 67)

On January 16, 2020, the first local COVID-19 cluster was reported in Sichuan. On January 24, 2020, a level-1 public health emergency response was launched, followed by the successive execution of multiple measures with respect to infection sources, routes of transmission and susceptible populations to effectively reduce population movement and gatherings and strengthen the management of close contacts. Main measures and the timeline are shown in Fig. 1.

On January 16, 2020, the first local COVID-19 cluster was reported. A total of 31 clusters were reported in January and 36 in February. The daily number of clusters peaked on January 30, 2020 (*n* = 7) and gradually declined after February 06, 2020. A total of 50 (74.63%) clusters were reported as of February 06, 2020. Temporal distribution of cluster reports is shown in Fig. 1. Among the clusters, 49 (73.13%) were caused by imported cases, and in 18 the infection source of the first case was unknown.

**Fig. 2:**
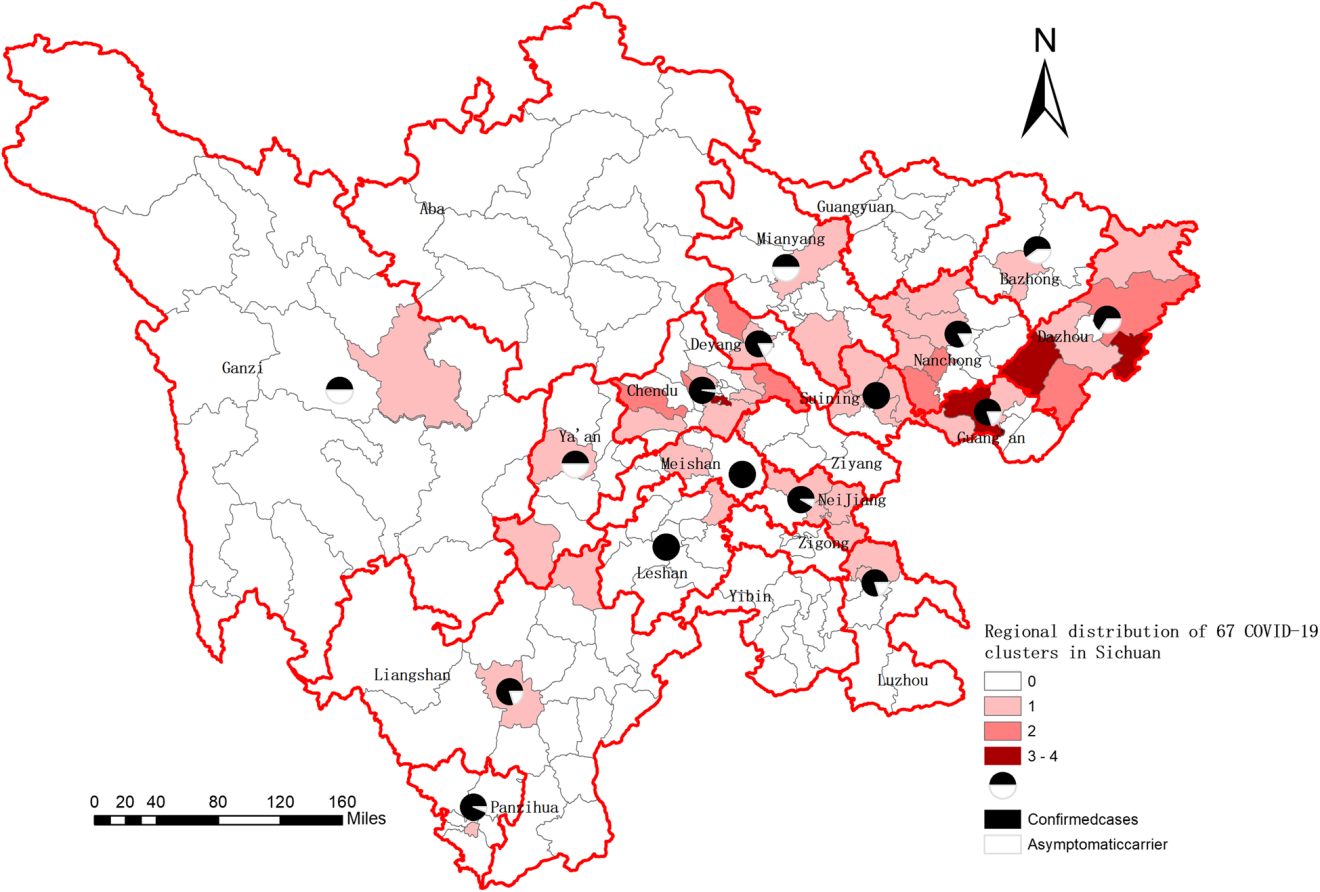
Regional distribution of 67 COVID-19 clusters in Sichuan. This figure was originally generated by using open access map layers available from National Geomatics Center of China (http://www.ngcc.cn/ngcc/html/1/index.html)

The 67 clusters had involved 16 prefectures and 48 districts/counties **(**Fig. 2**)**. Chengdu had the largest number of clusters (*n* = 17), followed by Dazhou (*n* = 12), Nanchong (*n* = 7) and Guang’an (*n* = 6). Clusters in the four prefectures accounted for 62.69% (42/67) of the total in the province.

**Fig. 3:**
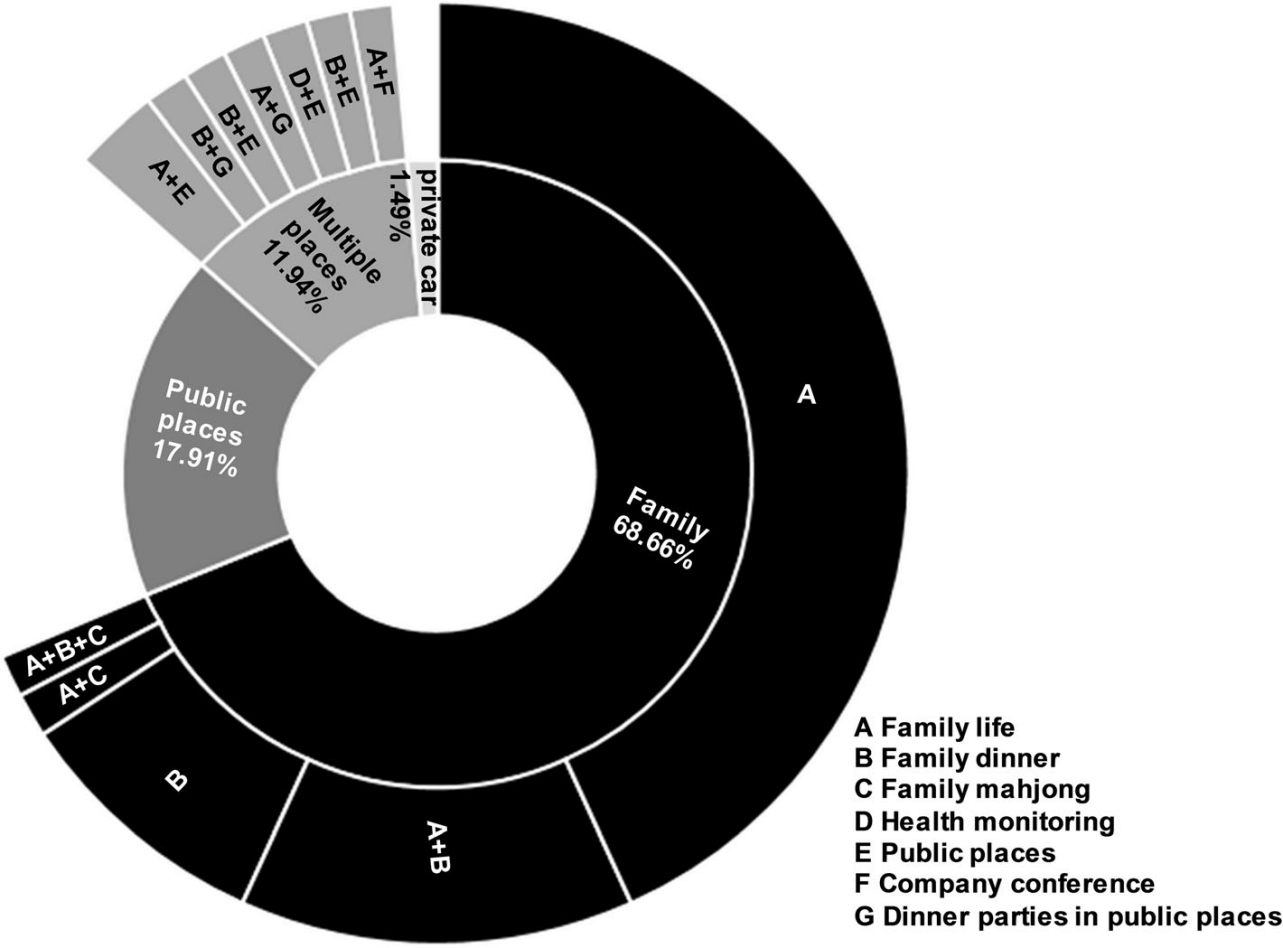
Distribution of exposure places and forms in clusters

Households were the primary exposure place in the reported clusters, accounting for 68.66% (46/67); living in the same household was the primary form of exposure, accounting for 60.87% (28/46). Clusters caused by exposure in multiple places and in multiple forms accounted for 11.94% (Fig. 3). Significantly more clusters were caused by family gatherings in February than in January (*p* = 0.002) (Table 1).

**Table 1:** Distribution of clusters caused by family gatherings in January and February

No. (%)	Total	Family dinner	Family life	Family life + family mahjong	Family life + family dinner	Family life + family dinner + family mahjong
January	15 (32.61)	1 (6.67)	11 (73.33)	1 (6.67)	2 (13.33)	0 (0.00)
February	31 (67.39)	6 (19.35)	17 (54.84)	0 (0.00)	7 (22.58)	1 (3.23)

### Characteristics of confirmed cases involved in clusters

In the 67 clusters, a total of 226 confirmed cases were reported, accounting for 42.01% (226/538) of the total in the whole province; among these confirmed cases, 164 were local confirmed cases resulting from clusters, accounting for 72.57% (164/226); the male/female ratio was 1:1, the mean age was 47.56 years (1 month-87 years), and patients 30 years of age or above accounted for 87.17% (197/226); involved were 75 (33.19%) G1 cases, 131 (57.96%) G2 cases and 20 (8.85%) G3 cases (Table 2); 23.01% (52/226) had a history of travel or residence in Wuhan, and 25.66% (58/226) had a history of travel or residence in other provinces except Wuhan. The constituent ratios of cases with a history of travel or residence in both Wuhan (*p <* 0.001) and other provinces except Wuhan (*p* = 0.006) were higher in G1 cases were higher in G2 and G3 cases, and the differences were statistically significant (Table 3).

**Table 2:** Distribution of confirmed cases in 67 clusters, by age and gender

	Total no.	Constituent ratio (%)	Case generation					
			G1 (no.)	Constituent ratio (%)	G2 (no.)	Constituent ratio (%)	G3 (no.)	Constituent ratio (%)
**Age group (yrs)**								
< 20	8	3.54	–	–	8	100	–	–
20-	21	9.29	2	9.52	17	80.95	2	9.52
30-	43	19.03	19	44.19	21	48.84	3	6.98
40-	59	26.11	24	40.68	32	54.24	3	5.08
50-	47	20.80	16	34.04	24	51.06	7	14.89
≥ 60	48	21.24	14	29.17	29	60.42	5	10.42
**Gender**								
Male	113	50.00	43	38.05	61	53.98	9	7.96
Female	113	50.00	32	28.32	70	61.95	11	9.73

**Table 3:** Travel or residence history of 226 confirmed cases

Case Generation	Cases (n)	Wuhan		Other Provinces except Wuhan	
		Cases (no.)	Constituent Ratio (%)	Cases (no.)	Constituent Ratio (%)
G1	75	45	60.00	29	38.67
G2	131	7	5.34	26	19.85
G3	20	–	–	3	15.00
		*χ*^*2*^ = 75.50	*p* < 0.001	*χ*^*2*^ = 10.16	*P* = 0.006
Total	226	52	23.01	58	25.66

**Fig. 4:**
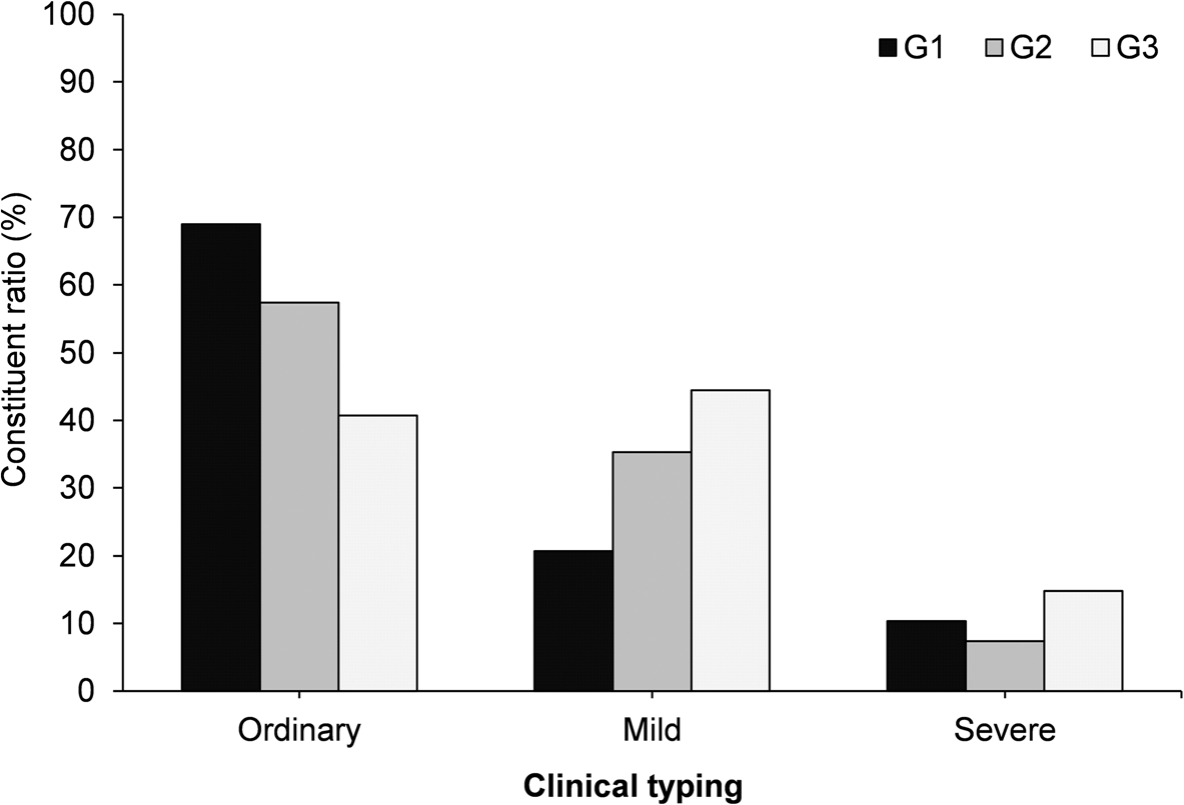
Clinical typing of confirmed cases (*n* = 226)

The 226 confirmed cases were clinically typed as follows: 65.04% (147/226) were ordinary, 26.55% (60/226) were mild, and 8.41% (19/226) were severe; no death occurred. With the increase of the transmission generation, the number of ordinary cases gradually decreased, with a significant difference noted overall (*p* = 0.045); a significant difference was found between G1 and G3 cases in the constituent ratio of ordinary cases (*p* = 0.013). No apparent between-generation difference was found in the constituent ratios of mild or severe cases (Fig. 4).

**Fig. 5:**
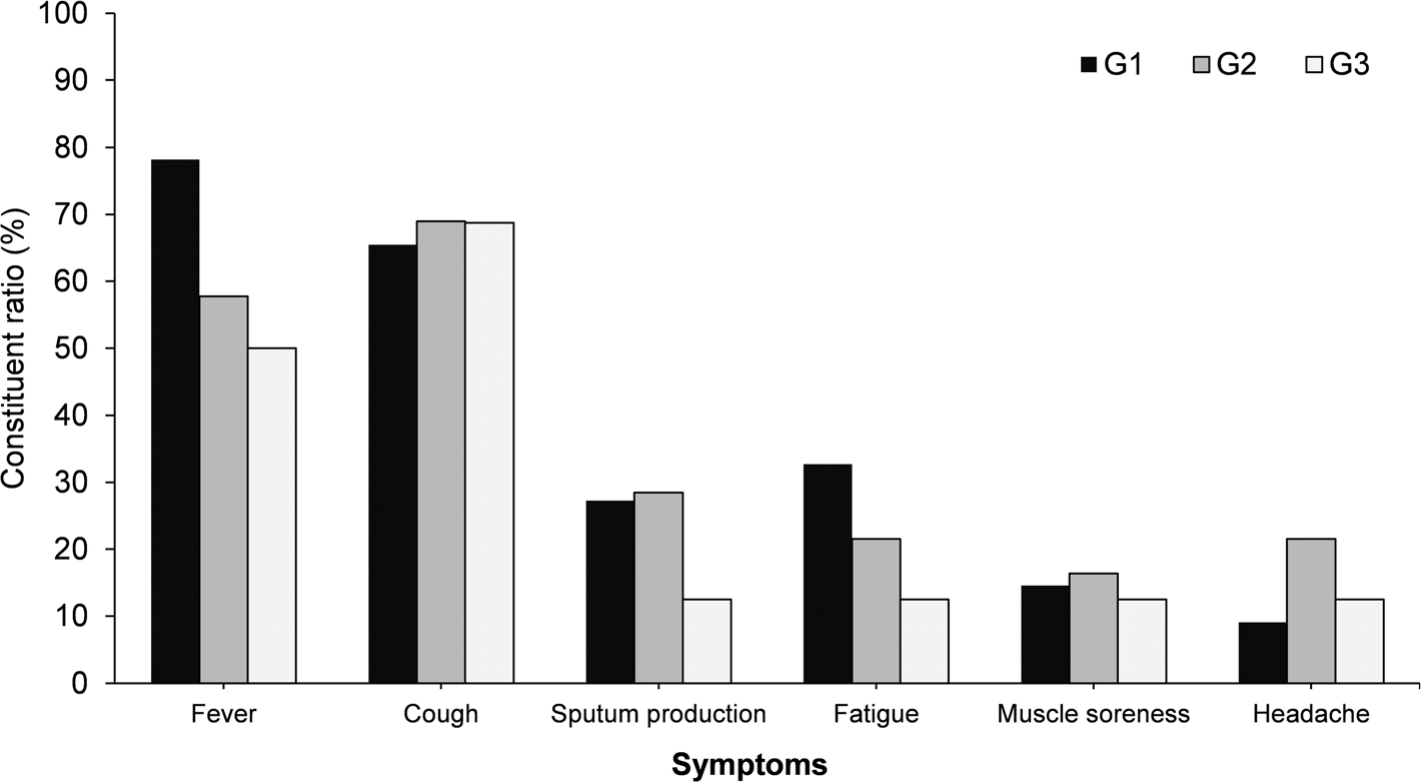
Main clinical symptoms of confirmed cases (*n* = 221)

Among the 226 cases, clinical information about 221 was complete. One hundred thirty-seven cases had fever, accounting for 61.99%, with a median maximum temperature of 38.09 °C (37.3–39.5 °C). Other main symptoms included dry cough, [66.52% (147/221)], sputum production [26.24% (58/221)], fatigue [25.34% (56/221)], sore throat [19.46% (43/221)] and headache [17.19% (38/221)]. Gastrointestinal symptoms were occasional, mainly including nausea [10.96% (24/221)] and diarrhea [6.79% (15/221)].The number of cases with fever showed a declining trend overall with the increase of transmission generation (*p* = 0.019), and significant differences were noted between G1 and G2 cases (*p* = 0.009) as well as G3 cases (*p* = 0.027) in terms of the constituent ratio of cases with fever. No statistically significant difference was found between generations in other symptoms (Fig. 5).

### Transmission characteristics

Among the 67 clusters, G2 cases were identified in 57 (85.07%) and G3 cases in 10 (14.93%); a total of 267 confirmed cases (including 41 asymptomatic carriers) were reported, and on average each cluster resulted in 3.99 (2–17) cases; the average attack rate was 8.54% (1.02–100%), and the average secondary attack rate was 6.11% (0.51–66.67%).

Starting from February 02, 2020, we expanded the time window of close contact tracing to cover 3 days prior to the appearance of clinical symptoms in primary confirmed cases or prior to the obtainment of a positive nucleic acid test in primary asymptomatic carriers. Attack rates and secondary attack rates before and after this time point were compared, and it was found that, following the expansion of the time window of close contact tracing, both the attack rate and the secondary attack rate increased, with statistical differences identified (Table 4).

**Table 4:** Comparison of attack rates and secondary attack rates in clusters before and after February 02, 2020^a^

Time	No. of cases	No. of exposed persons	Attack rate (%)	***p*** value	No. of secondary cases	No. of exposed persons	Secondary attack rate (%)	***p*** value
Before February 02, 2020	156	2027	7.70	0.022	107	1990	5.38	0.022
After February 02, 2020	111	1100	10.09		80	1072	7.46	

^a^Both the number of cases and the number of secondary cases include confirmed cases and asymptomatic carriers

### Timing of key events

Among the 226 cases, 78.76% (178/226) had been exposed to confirmed cases. In 82.02% (146/178) of such cases, the exposure to the confirmed cases occurred at or after onset of the disease in them. In 10 cases (involved in 9 clusters), the exposure occurred before the confirmed cases they contacted with developed clinical symptoms, and they had been exposed only once while the possibility of exposure to other infection sources could be ruled out. The median time of exposure was 3 d (1–4 d) prior to disease onset in the confirmed cases they contacted with, and the mean was 2.5 d; 90.00% (9/10) of the cases were exposed within 3 d prior to disease onset in the confirmed cases they contacted with.

Of 226 confirmed cases, the median interval between hospital visit and onset was 1 d (0–23 d), the median interval between confirmation and onset was 5 d (0–27 d), and the median interval between isolation and confirmation was − 2 d (− 19–0 d). Twenty-eight cases met the criteria for incubation period determination (involved in 17 clusters). The average incubation period was 10.3 d, the median incubation period was 8.5 d (1–24 d) and the incubation period was > 14 d in 21.43% (6/28).

### Asymptomatic carriers

In the 67 clusters, a total of 41 asymptomatic carriers (all were identified during medical observation of close contacts) were reported, including 23 males and 18 females. The mean age was 35.31 years (8 months-81 years); and patients 30 years of age or above accounted for 70.73% (29/41). Involved were 8 (19.51%) G1 cases, 31 (75.61%) G2 cases and 2 (4.88%) G3 cases.

In one cluster, one G1 case (severe) was found to have spread the disease to one G2 case (mild) and one G3 case (asymptomatic). Two clusters were caused by asymptomatic carriers and, in both clusters, only G2 cases developed, including one (asymptomatic) to two (confirmed) transmission in one cluster and one (asymptomatic) to one (confirmed) transmission in another cluster. The possibility of other infections was ruled out in all G2 cases. By the end of the two clusters, the G1 asymptomatic carriers had not presented with clinical symptoms or imaging abnormalities.

## Discussion

Our analysis of COVID-19 clusters in Sichuan reveals that the majority of clusters were reported before February 06 and occurred in four regions of Sichuan, and three-fourth of the clusters were caused by importations from other provinces including Wuhan. The study also found that the proportion of cases with a history of travel or residence in Wuhan was significantly higher in G1 cases than in secondary cases and the clusters mainly occurred in households with a greater proportion in February than in January. These characteristics were similar to clusters in other provinces in China [3].

The above characteristics of COVID-19 clusters were supposed to be related with several factors. Firstly, the epidemic happened during the Spring Festival in China, when large numbers of people in China returned home for family reunion or visited their friends or relatives, forming a population shift peak, particularly in the mid-late January. Secondly, Chengdu, the capital of Sichuan Province, as a rapidly growing metropolis in West China, has well-developed transport infrastructure including highways, railways and airlines, which facilitated the spread of the disease [4]; big data analysis also showed that Chengdu was ranked the top in terms of inflow of people from Wuhan [5–7]; Dazhou and Guang’an, located in the northeast of Sichuan, are adjacent to Chongqing, which was relatively hardly hit by the epidemic among provinces other than Hubei in China [8]. Finally, on January 24, 2020, Sichuan launched a level-1 public health emergency response [9, 10], followed by the successive execution of multiple measures with respect to infection sources, routes of transmission and susceptible populations, effectively reducing population movement and gatherings and strengthening the management of close contacts. Specifically, on January 25, 2020, the Administration for Market Regulation, the Health Commission and the Department of Commerce of Sichuan Province jointly issued a document [11] for strengthening the supervision of dinner gatherings; on January 28, 2020, the People’s Government of Sichuan Province issued an order [12] requiring suspension of massive gatherings and business of recreational places and banning dinner gatherings. On February 8, 2020, the provincial headquarters for emergency response issued a notice on strengthening the supervision of dinner gatherings in rural areas [13], banning dinner gatherings involving two or more families. The above measures have effectively reduced people’s gatherings including festival celebrations and company dinner parties. However, still a minority of people, following traditional customs, organized and participated in family dinners and entertainment activities with inadequate protection during the Chinese New Year, resulting in the familial clustering characteristic of the epidemic.

The attack rate of COVID-19 clusters in our study was slightly lower than 9.6% in a study located in Shenzhen [14]. The interval between hospital visit and onset was shorter than that reported in Hunan [15]; related data were not available from other provinces. The study also showed that the attack rate and the secondary attack rate were significantly higher after February 02, 2020 than before; cases were mainly confirmed during the isolation period and asymptomatic carriers were identified in the management of close contacts, which suggested that the intensive close contact management measures in Sichuan including expansion of the close contact tracing time window to cover 3 d prior to the presence of clinical symptoms (or positive test for asymptomatic carriers) in the confirmed cases they contacted with, implemented from February 02, 2020, and the conduct of nucleic acid testing at both the start and the end of quarantine of close contacts, implemented from February 11, 2020, might have contributed to the discovery and control of cases and the reduction of spread.

The median incubation period in the limited cases in Sichuan was corresponding to the finding in other studies in China that the incubation period in the majority of cases was < 14 d, but being longer than the median incubation period reported in other studies (3–7 d) [16–20]. It was also found that the proportion of cases with an incubation period > 14 d was higher than that reported in a study in Henan (7.45%) [21]. Ten cases exposed before the confirmed cases they contacted with presented with clinical symptoms, of which most cases exposed within 3 d prior to disease onset, and these cases had been exposed only once and other potential sources of infection could be ruled out, suggesting COVID-19 is possibly transmittable during the incubation period, similar to a report in Zhejiang [22]. At the same time, the majority of cases involved in the clusters exposed within 3 days prior to onset of the disease in confirmed cases they contacted with, suggesting COVID-19, like influenza, is possibly contagious at the end of the incubation period or tracing back to 3 d prior to disease onset in seeking close contact could miss earlier infections in the incubation period. What’s more, two clusters caused by asymptomatic carriers indicated COVID-19 could be transmitted by asymptomatic infected persons, as is similar to the report in Henan [21]. Nevertheless, the asymptomatic carriers had only caused a small number of G2 cases, indicating the transmissibility in this population may be limited. The above two populations cannot be effectively identified and thus are of special public health significance for sustained transmission of COVID-19 [23]; due to limited available data, the transmissibility of the disease cannot be analyzed, and studies on its pathogenicity and transmissibility in these populations are awaited.

Main presentations of confirmed cases involved in the clusters in Sichuan included fever, respiratory and systemic symptoms, basically corresponding to the reported clinical characteristics of COVID-19 cases, and no characteristic clinical symptom was seen [18, 24–27]. In clusters, the proportion of ordinary cases and cases with fever decreased with the increase of the case generation, suggesting that the severity of clinical symptoms might gradually lighten with the development of the epidemic. This corresponded to reports that, in the initial period of the epidemic, cases were mainly severe and the case fatality rate was high [18] and, in the later period of the epidemic, cases were mainly mild [26]. This is possibly because the virus will maintain moderate virulence during passage and evolution for the purpose of long-time reproduction in human and the infection was limited to the upper respiratory tract [28]. In addition, it was reported that antibody levels would not last long, and repeated infection was common [29, 30]. However, limited data are available and further studies on COVID-19 antibody levels and protective effects are awaited for investigating whether post-infection antibody levels will decline following the mitigation of COVID-19 clinical symptoms or whether repeated infection will occur as a result of the short-term maintenance of antibody levels.

The findings in the study are subject to several limitations. On the one hand, as only cases exposed only once were included, the cases calculated for the incubation period was limited. On the other hand, PCR detection of respiratory tract specimens was carried out once each time when close contacts were isolated and released, and the isolation period was 14 days. If an asymptomatic carrier recovered spontaneously during the isolation period, the attack rate might be underrated due to the missing count of cases.

## Conclusions

In conclusion, our study demonstrated the comprehensive epidemic situation of COVID-19 in Sichuan Province and confirmed the infectivity during the incubation period and asymptomatic infection, providing a reference for decision makers to formulate and adjust control measures.

## Data Availability

The datasets used and/or analysed during the current study are available from the corresponding author on reasonable request.

## Abbreviations

WHO: World health organization
COVID-19: Coronavirus disease
RR: Respiratory rate
SpO2: Pulse oxygen saturation
PaO2: Arterial partial pressure of oxygen
FiO2: Fraction of inspiration O2
G1: First generation
G2: Second generation
G3: Third generation
PCR: Polymerase chain reaction
